# Population Pharmacokinetic Modeling and Dose Optimization of Vancomycin in Chinese Patients with Augmented Renal Clearance

**DOI:** 10.3390/antibiotics10101238

**Published:** 2021-10-12

**Authors:** Sixuan Zhao, Na He, Yahui Zhang, Chuhui Wang, Suodi Zhai, Chao Zhang

**Affiliations:** 1 Department of Pharmacy, Peking University Third Hospital, Beijing 100191, China; zhao_sixuan@126.com (S.Z.); hn12141122@163.com (N.H.); zhangyahui61@126.com (Y.Z.); chuhui_wang@163.com (C.W.); 2 Department of Pharmacy Administration and Clinical Pharmacy, School of Pharmaceutical Science, Peking University, Beijing 100191, China; 3 Department of Pharmacy, Beijing Tongren Hospital, Capital Medical University, Beijing 100730, China

**Keywords:** area under the curve, augmented renal clearance, dosage regimens, population pharmacokinetic model, vancomycin

## Abstract

Patients with augmented renal clearance (ARC) have been described as having low vancomycin concentration. However, the pharmacokinetic model that best describes vancomycin in patients with ARC has not been clarified. The purpose of this study is to determine the pharmacokinetic of vancomycin in Chinese adults and the recommend dosage for patients with different renal function, including patients with ARC. We retrospectively collected 424 vancomycin serum concentrations from 209 Chinese patients and performed a population pharmacokinetic model using NONMEM 7.4.4. The final model indicated that the clearance rate of vancomycin increased together with the creatinine clearance, and exhibited a nearly saturated curve at higher creatinine clearance. The estimated clearance of vancomycin was between 3.46 and 5.58 L/h in patients with ARC, with 5.58 being the maximum theoretical value. The central volume of distribution increased by more than three times in patients admitted to Intensive Care Unit. Monte Carlo simulations were conducted to explore the probability of reaching the target therapeutic range (24-h area under the curve: 400–650 mg·h/L, trough concentration: 10–20 mg/L) when various dose regimens were administered. The simulations indicated that dose should increase together with the creatinine clearance until 180 mL/min. These findings may contribute to improving the efficacy and safety of vancomycin in patients with ARC.

## 1. Introduction

Vancomycin is a glycopeptide antibiotic used to treat a number of gram-positive infections [1]. It is the first-line choice of treatment for infections caused by methicillin-resistant *Staphylococcus aureus* (MRSA).

Vancomycin is excreted in urine by glomerular filtration, without appreciable metabolism [2]. The clearance rate is mainly affected by renal function. Dosage must be adjusted for patients with renal dysfunction because the risk of toxicity is increased by high blood concentrations [3]. At the same time, patients with augmented renal clearance (ARC) have lower concentrations of vancomycin, leading to suboptimal drug exposure and treatment failure [4,5]. A longer length of stay in the Intensive Care Unit has also been reported [6,7]. Of note, the administration of subtherapeutic doses increased the prevalence of antibiotic resistance [8].

Individualized dosage regimen is therefore necessary for safe and effective administration. Establishing a population pharmacokinetic model is optimal for planning dosing regimen. However, most of the studies provided only regimens for patients with impaired renal function [9,10,11,12]. The pharmacokinetic profile of vancomycin in patients with ARC has not been well established and an appropriate dosing recommendation is very necessary.

We therefore conducted a population pharmacokinetic study of vancomycin to estimate its variability in patients with abnormal renal function, including patients with ARC.

## 2. Results

### 2.1. Patients and Data Collection

We retrospectively collected and analyzed 424 vancomycin serum concentrations from 209 Chinese patients. Among these, 82 (39.2%) were admitted at the Intensive Care Unit (ICU) and 127 (60.8%) were admitted at other departments. A total of 40 (48.8%) ICU patients and 6 (2.9%) non-ICU patients developed shock or evidence of multiple organ failure. 51 (24.4%) patients had a creatinine clearance (CL_cr_) ≥ 130 mL/min. Vancomycin was administered at different dosages (median daily dosage 1875 mg), and mostly evaluated between 5 and 12 h after administration. Clinical characteristics of enrolled patients are displayed in Table 1.

### 2.2. Population Modeling

According to the goodness-of-fit (GOF) plots and Akaike information criterion (AIC) values, a two-compartment model (AIC, 2089.498) was more appropriate to describe the pharmacokinetic profile than a one-compartment model (AIC, 2162.859). The inter-individual variability (IIV) was successfully estimated for clearance (CL) and central volume of distribution (V_c_). The CL_cr_ and the admission at the ICU were identified as significant covariates for CL and V_c,_ respectively, in the forward addition and backward elimination procedures. The final model was therefore developed to describe the concentration-time profile of vancomycin (Equations (1)–(4)):

(1) $$\mathrm{CL}=\frac{5.58\;*\;{\mathrm{CG}}_{i}{}^{1.5}}{\left(93.8^{1.5}+\;{\mathrm{CG}}_{i}{}^{1.5}\right)}*e^{\eta_{1}}\;(L/h),$$

(2) $$V_{c}=8.02\;(\mathrm{non}-\mathrm{ICU}\;\mathrm{patients})\;\mathrm{or}\;35.7\;(\mathrm{ICU}\;\mathrm{patients})\;*\;e^{\eta_{2}}\;(L),$$

Q = 2.66 (L/h),(3)

V_p_ = 36.8 (L), (4)

where CG is the creatinine clearance estimated by Cockcroft-Gault equation, while 5.58 is the theoretical maximum clearance of vancomycin in patients with creatinine clearance between 18.4 and 390.7 mL/min. When the creatinine clearance is 93.8 mL/min, the drug clearance is at 50% of its maximum value. The steepness parameter is 1.5. V_c_ is 8.02 L and 35.7 L for non-ICU and ICU patients, respectively. The typical value of the peripheral volume of distribution (V_p_) is 36.8 L. η_1_ and η_2_ represent the interindividual variations of CL and V_c_, respectively.

### 2.3. Model Evaluation

**Figure 1:**
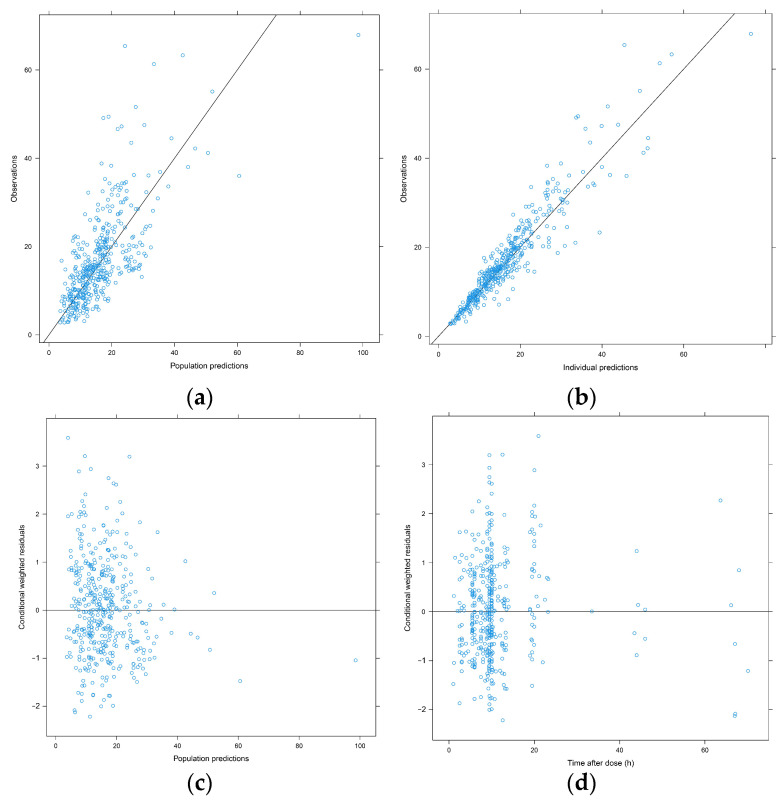
Goodness-of-fit plots of vancomycin population pharmacokinetic model. (**a**) observed concentration versus population prediction (PRED); (**b**) observed concentration versus individual prediction (IPRED); (**c**) conditional weighted residuals (CWRES) versus PRED; (**d**) CWRES versus time after dose (TAD).

**Figure 2:**
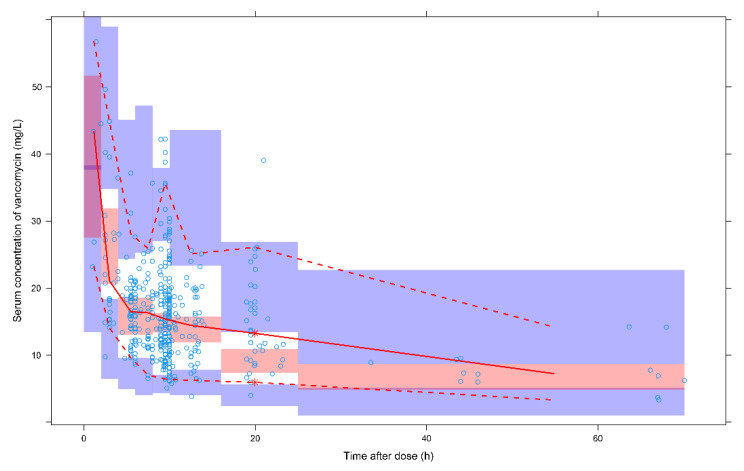
Prediction corrected-visual predictive check (pc-VPC) plot of the final model. Blue circles: observed vancomycin concentrations. Red solid and dashed lines: the 50th, 5th, and 95th percentiles of the observed concentrations. 3 shaded areas: the 90% CIs of the 5th, 50th, and 95th percentiles of the simulated concentrations.

The GOF plots indicate that the final model predictions agreed with the observed plasma concentration of vancomycin. Observed data versus either the population or the individual predicted values were closely distributed around the y = x line (Figure 1a,b). The conditional weighted residuals (CWRES) were approximately 0, randomly and homogeneously distributed (Figure 1c,d). A success rate of 84.6% was obtained from the bootstrap analysis. The estimated model parameters were within the bootstrap confidence interval (Table 2). The pc-VPC plot suggested that the simulated models were consistent with the observed values (Figure 2).

### 2.4. Dosage Recommendation

The probability of AUC_24_ between 400 and 650 mg·h/L at several regimens was calculated using the final model (Figure S1). The dosage regimen providing the highest target attainment rate determined the optimal regimen. The estimated AUC_24_ at our regimen was shown in violin plot (Figure S2). The dose of vancomycin increased together with CL_cr_ until 180 mL/min. The recommended dosage regimens for targeted AUC_24_ were summarized in Table 3. The recommended regimens for targeted C_t_ are described in Supplement Table S1. The probability of target attainment (PTA) was between 41 and 67% in patients with different renal function.

## 3. Discussion

Our results suggest that the CL_cr_ and ICU admissions influence the pharmacokinetics of vancomycin. Patients with ARC (CL_cr_ ≥ 130 mL/min) showed between 1.3 and 2.1 times higher drug clearance than patients with normal kidney function. The central volume of distribution increased by 3.5 times in ICU patients, compared with non-ICU patients. We therefore designed an individualized dosing regimen based on these two covariates.

We identified the CL_cr_ calculated with the Cockcroft-Gault equation as the most significant covariate that affected the elimination of vancomycin. The drug clearance rate increased together with CL_cr_ in a saturation curve, with the theoretical maximum clearance being 5.58 L/h. In previous pharmacokinetic studies, the clearance rate constantly increased [13,14,15], whereas Chu et al. found that the trend is weaker in patients with ARC [16]. Consistently, a similar trend was plotted into a scatter diagram format with CL_cr_ and the clearance (Supplement Figure S3). We therefore tried to fit a saturation correlation and found lower objective function value (OFV) of the model (OFV 1843.222) compared with linear (OFV 1864.24), exponential (OFV 1929.264) and power (OFV 1856.897) models, indicating that the correlation was more consistent with the actual relationship.

Although different models have been implemented regarding vancomycin pharmacokinetics, little is known about the behavior in patients with ARC [17]. In observational cohort studies, a lower blood concentration has been described in patients with ARC [18,19]. Our model suggested between 1.3 and 2.1 times higher clearance in patients with ARC than in patients with normal kidney function. Another study reported a clearance rate of 8.52 L/h, much higher than what we observed [20]. The age was used as a covariate and might explain their findings, obtained in a younger population. 

The apparent volume of distribution increased by more than three times in ICU patients (35.7 L), compared with non-ICU patients (8.02 L). Previous studies reported similar results [21,22,23]. Several pathophysiological changes might be related to PK modifications in ICU patients [24]. Intravenous fluid loading, hypoalbuminemia and endothelial damage may increase capillary permeability and contribute to interstitial space expansion in the critically ill, especially in cases of sepsis and septic shock [25]. Hydrophilic drugs, characterized by a distribution limited to the extracellular space, are significantly affected [24]. A recent study suggested that an increased volume of distribution (V_d_) may be due to sepsis-induced third space losses [26]. In accordance, we found that more ICU patients received a diagnosis of shock or multiple organ failure. Although the two diagnoses were not identified as significant covariates of V_d_ in our study, we cannot refuse the influence of these diseases on V_d_. The proportion of patients with corresponding diagnosis is relatively low, which may interfere with the identification of influencing covariates. On the other hand, although not been diagnosed, ICU patients with different degrees of sepsis (sepsis, severe sepsis and septic shock) are associated with varying degrees of fluid retention.

A larger V_d_ of hydrophilic drugs has been reported in ICU patients, but the clinical relevance is questionable. An aminoglycoside is supposed to have lower concentrations in patients with higher V_d_ and therefore needs an augmented dose [24,25]. An increased dosing is also necessary for β-lactam antibiotics to prevent visible growth of a microorganism [27,28]. However, an increased V_d_ may not have a significant effect on the maintenance dose of vancomycin, as the AUC depends only on the clearance rate at the steady state. At the same time, the increased V_d_ might improve the penetration of the drug, which is helpful against certain infections. Higher loading and daily doses were suggested by some authors [29]. An augmented loading dose may increase the body’s exposure to vancomycin in the initial treatment phase and may be helpful for the early elimination of the bacterium in vivo.

We recommend a dosage regimen for patients with a CL_cr_ between 15 and 180 mL/min. Patients with a CL_cr_ > 180 mL/min do not need to increase the dose, which is consistent with the saturation correlation dimension of our model. Other studies reported higher daily doses than ours [9,12,30]. Differences in the choice of simulation target might be the reason. The daily dose was higher when the trough concentration was used as the simulation target (Supplement Table S1). However, the AUC is more recommended as may reduce the occurrence of vancomycin-associated acute kidney injury [31,32]. A meta-analysis demonstrated that AUC/minimum inhibitory concentration = 400 is a reasonable target of mortality and infection treatment failure [33]. An AUC_24_ > 650 mg·h/L was associated with a higher risk of nephrotoxicity [34]. We therefore selected AUC_24_ between 400 and 650 mg·h/L as the target in this study [35]. 

Our study has some limitations. First, the sample size was limited. Other clinical data could help to characterize the differences between ICU and non-ICU patients, including the covariate Acute Physiology and Chronic Health Evaluation II or other scores. In the future, prospective studies with a larger sample size may be helpful to verify the performance of the model, as well as the effectiveness of the proposed regimens in real-world patients.

## 4. Materials and Methods

### 4.1. Patients and Data Collection

This study was approved by Peking University Third Hospital (PUTH) Ethics Committee (reference number M2020377). Data from hospitalized patients at PUTH between January 2010 and June 2018 were retrospectively collected. Age, gender, total body weight (TBW), department, primary diagnosis, vancomycin dosage, serum creatinine (SCr) and serum vancomycin concentration were recorded. Patients were eligible to participate in the study if they: (i) aged ≥ 18 years; (ii) received intermittent intravenous vancomycin therapy; (iii) had data recorded, including age, gender, TBW, SCr and at least one serum vancomycin measurement. Patients were excluded from the study if they: (i) had been diagnosed with end-stage renal disease, including patients with CL_cr_ < 15 mL/min or receiving renal replacement therapy; (ii) had been diagnosed with acute kidney injury before or during treatment; (iii) had been admitted to the Hematology Department or the Surgical Department of the Intensive Care Unit; (iv) had been pre-treated with vancomycin in other hospitals; (v) had significant missing data. 

### 4.2. Evaluation of Serum Vancomycin Concentration

Vancomycin hydrochloride (abbreviated as vancomycin) for intravenous administration was obtained from Eli Lilly and Company and Zhejiang Pharmaceutical Co., Ltd. The serum concentration of vancomycin was determined by commercial chemiluminescent microparticle immunoassay (CMIA) assay using the ARCHITECT platform with the ARCHITECT iVancomycin assay obtained from Abbott Laboratories Trading Co., Ltd. (Shanghai, China).

### 4.3. Population Pharmacokinetic Modeling

The model estimation was performed using NONMEM 7 software (version VII, level 4.4; ICON Development Solutions, Ellicott City, MD, USA) with the FOCEI method. Analysis and post-processing were performed with the PsN toolkit and Xpose4 (version 4.6.1) through the statistical package R.

Classical one- and two-compartment models were fitted to the data. An exponential model was used to estimate the interindividual variability of the pharmacokinetic parameters (Equation (5)). The residual errors were described by a constant coefficient of variation model (Equation (6)). AIC values, GOF plots and the numerical estimates were used to determine the structure model.

P_i_ = P_pop_ ∗ e^η^, (5)

where P_pop_ and P_i_ represent the pharmacokinetic parameters for the population and each individual, respectively. η is a random variable for each individual following a normal distribution with a mean of 0 and a variance of ω^2^.

C_obs_ = C_pred_ + C_pred_ ∗ ε, (6)

where C_pred_ and C_obs_ represent the predicted concentration and the observed vancomycin concentration in the serum, respectively. ε represents the proportional error assumed to follow a normal distribution with a mean of 0 and a variance of σ^2^.

Factors considered in relation to the pharmacokinetics of vancomycin were age, gender, TBW, department, primary diagnosis and CL_cr_ estimated according to the Cockcroft-Gault equation. An exploratory graphical analysis was performed to identify characteristics that may influence pharmacokinetic parameters. The covariates showing a correlation with pharmacokinetic parameters were introduced into the model sequentially. The significance of the covariates was calculated through the OFV. An OFV decrease of more than 3.84 (*p* < 0.05) was considered statistically significant during the forward inclusion process. All of the significant covariates were incorporated in the full model and then excluded from the model one at a time. An OFV increase of more than 10.83 from the full model (*p* < 0.001) was considered statistically significant.

### 4.4. Model Validation

Validation was performed by GOF plots, bootstrap and prediction corrected-visual predictive check (pc-VPC) approaches. GOF plots illustrated the overall performance of the model. The bootstrap median values and 95% confidence intervals for each estimate were compared with those from the original dataset. The pc-VPC approach was applied to determine whether sample data were consistent with the 90% prediction interval of 1000 simulated datasets from the final model.

### 4.5. Simulation and Dose Optimization

The Monte Carlo simulations (N = 10,000) were performed for each renal function classification (i.e., 15–29 mL/min, 30–44 mL/min, 45–59 mL/min, 60–89 mL/min, 90–119 mL/min, 120–149 mL/min, 150–179 mL/min, 180–209 mL/min, 210–239 mL/min, 240–269 mL/min, and 270–299 mL/min). Dosing intervals were set at 8, 12 or 24 h with 250–2500 mg per dose. The probability of target (steady state 24-h area under the curve (AUC_24_): 400–650 mg·h/L, steady state trough concentration (Ct): 10–20 mg/L) attainment was calculated. The dosage regimens with the highest PTA were recommended.

## 5. Conclusions

The current study established a population pharmacokinetic model for vancomycin in adult patients with different renal function, including patients with ARC. An initial dosing regimen of vancomycin was proposed for patients with insufficient, normal and augmented renal clearance.

## Figures and Tables

**Table 1:** Demographic characteristics of patients.

Characteristic	Patients (N = 209)
Male, n (%)	126 (60.3%)
ICU, n (%)	82 (39.2%)
CL_cr_ ≥ 130 mL/min, n (%)	51 (24.4%)
Shock, n (%)	39 (18.7%)
MOF, n (%)	7 (3.3%)
Age, years (mean ± SD)	66.0 ± 16.4
TBW, kg (mean ± SD)	63.4 ± 12.9
Mean CL_cr_^a^, mL/min (median (range))	86.7 (18.4–390.7)
Number of samples, n	424
Number of samples per patient, n (%)	
1	103 (49.3%)
2	50 (23.9%)
3	29 (13.9%)
≥4	27 (12.9%)
Mean daily dosage, mg (median (IQR))	1875.0 (1461.9–2352.0)
Number of samples collected after the start of infusion, n (%)	
<3 h	25 (5.9%)
3–5 h	16 (3.8%)
5–12 h	294 (69.3%)
12–24 h	75 (17.7%)
>24 h	14 (3.3%)
Mean concentration after the start of infusion, mg/L	
<3 h	29.5
3–5 h	22.9
5–12 h	16.5
12–24 h	15.3
>24 h	10.3

ICU, Intensive Care Unit; CL_cr_, creatinine clearance calculated by the Cockcroft–Gault equation; MOF, multiple organ failure; SD, standard deviation; TBW, total body weight; IQR, interquartile range; ^a^, the mean value of CL_cr_ for each patient during admission.

**Table 2:** Population parameter estimates of the base and final population models.

Parameter	Base Model Estimate (RSE%)	Final Model Estimate (RSE%)	Bootstrap Median (95% CI)
CL (L/h)	2.56(7%)	-	-
CL_max_ (L/h)	-	5.58 (17%)	5.58 (4.26–8.52)
CG_CLmax50_ (L/h)	-	93.8 (24%)	94.65 (66.99–178.63)
s	-	1.5 (14%)	1.49 (1.16–1.95)
V_c_ (L)	13.1 (11%)	-	-
V_c non-ICU_ (L)	-	8.02 (12%)	7.90 (4.90–11.80)
V_c ICU_ (L)	-	35.7 (13%)	36.66 (26.68–48.37)
Q (L/h)	4.9 (26%)	2.66 (12%)	2.64 (1.80–3.58)
V_p_ (L)	40.2 (13%)	36.8 (15%)	36.26 (25.51–49.36)
IIV CL	0.319 (12%)	0.0771 (16%)	0.075 (0.05–0.10)
IIV V_c_	1.65 (39%)	0.223 (56%)	0.20 (0.0039–0.53)
Additive residual error	0.0479 (18%)	0.0466 (14%)	0.045 (0.032–0.060)

CL, clearance; CL_max_, theoretical maximum clearance; CG_CLmax50_, value of creatinine clearance reaching 50% of the maximum drug clearance; s, steepness parameter; V_c_, distribution volume of the central compartment; V_c non-ICU_, V_c_ of non-ICU patients; V_c ICU_, V_c_ of ICU patients; Q, inter-compartment clearance; V_p_, distribution volume of the peripheral compartment; IIV, inter-individual variability; RSE, relative standard error; CI, confidence interval.

**Table 3:** Recommended initial dosage regimens for targeted AUC_24_ between 400 and 650 mg·h/L.

CL_cr_ (mL/min)	Dosage	PTA (%)
15–29	250 mg Q24 h	41.44
30–44	500 mg Q24 h	53.69
45–59	750 mg Q24 h	57.64
60–89	1250 mg Q24 h	57.32
90–119	750 mg Q12 h	61.58
120–149	1750 mg Q24 h	62.33
150–179	1000 mg Q12 h	62.56
≥180	750 mg Q8 h	61.69

CL_cr_, creatinine clearance; PTA, probability of steady-state AUC_24_ between 400 and 650 mg·h/L.

## Data Availability

The data presented in this study are available on request from the first/corresponding author. The data are not publicly available due to confidentiality agreement with Peking University Third Hospital.

## Supplementary Materials

The following are available online at https://www.mdpi.com/article/10.3390/antibiotics10101238/s1, Figure S1: Probability attainment for targeted AUC_24_ 400–650 mg·h/L., Figure S2. Distribution of AUC_24_ at recommended vancomycin regimens., Figure S3. Scatter diagram format with creatinine clearance calculated by the Cockcroft–Gault equation (CG) and the drug clearance (Clearance)., Table S1: Recommended initial dosage regimens for targeted trough concentration of 10–20 mg/L attainment.
