# Supplementary material for: Population Pharmacokinetic Modeling and Dose Optimization of Vancomycin in Chinese Patients with Augmented Renal Clearance
Source: Antibiotics (Basel). 2021 Oct 12;10(10):1238. doi: 10.3390/antibiotics10101238 (PMC8532702; doi:10.3390/antibiotics10101238)
Supplement: Supplementary file 1 [file antibiotics-10-01238-s001.zip › antibiotics-1378074-supplementary.pdf]

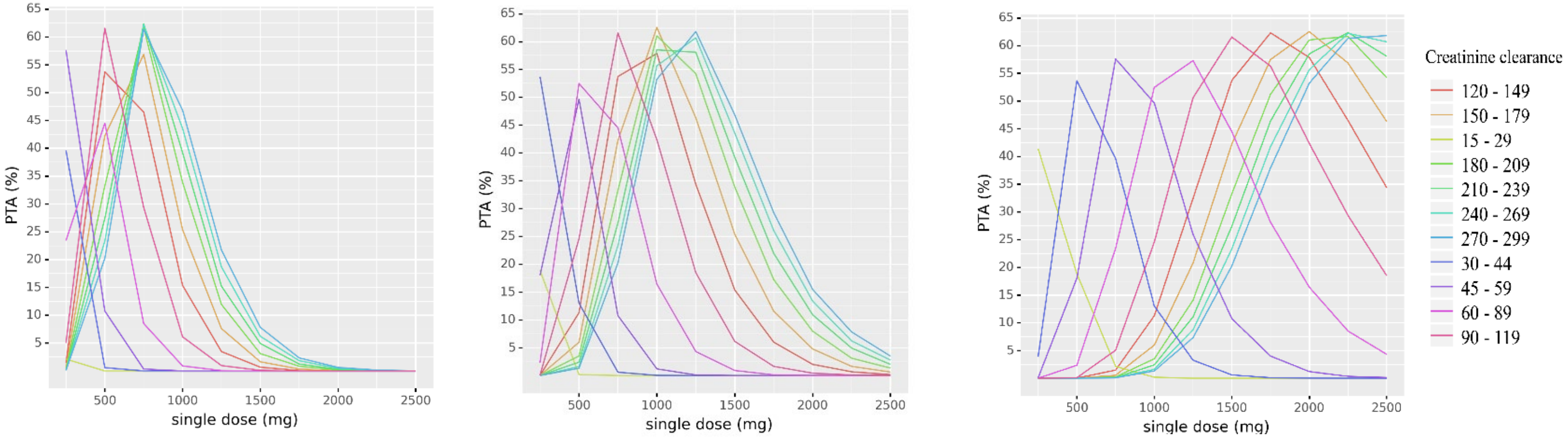

(**a**) (**b**) (**c**)

Figure S1. Probability attainment for targeted AUC_{24} 400-650 mg·h/L with dose interval of 8h (a); 12h (**b**) and 24h (**c**).

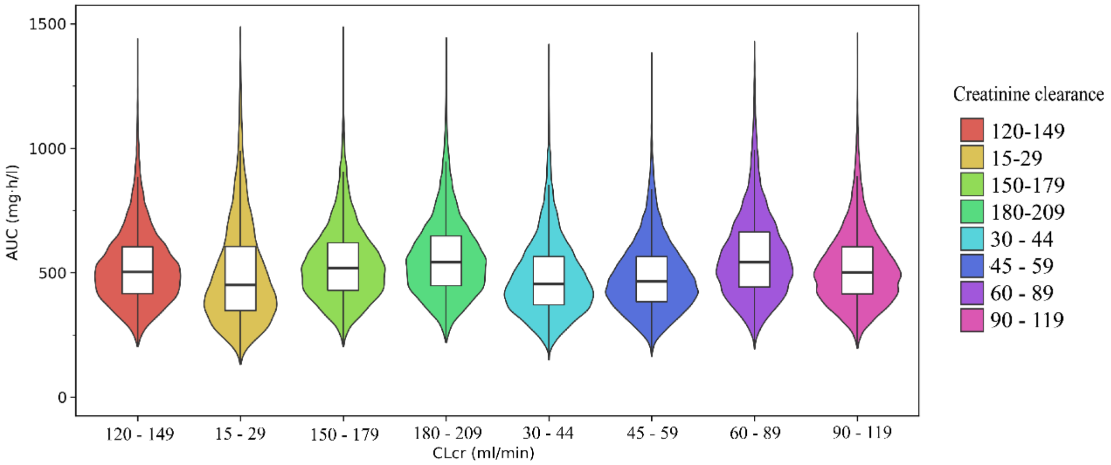

Figure S2. Distribution of AUC_{24} at recommended vancomycin regimens. Central mark, bottom and top edges: the median, 25th and 75th percentiles.

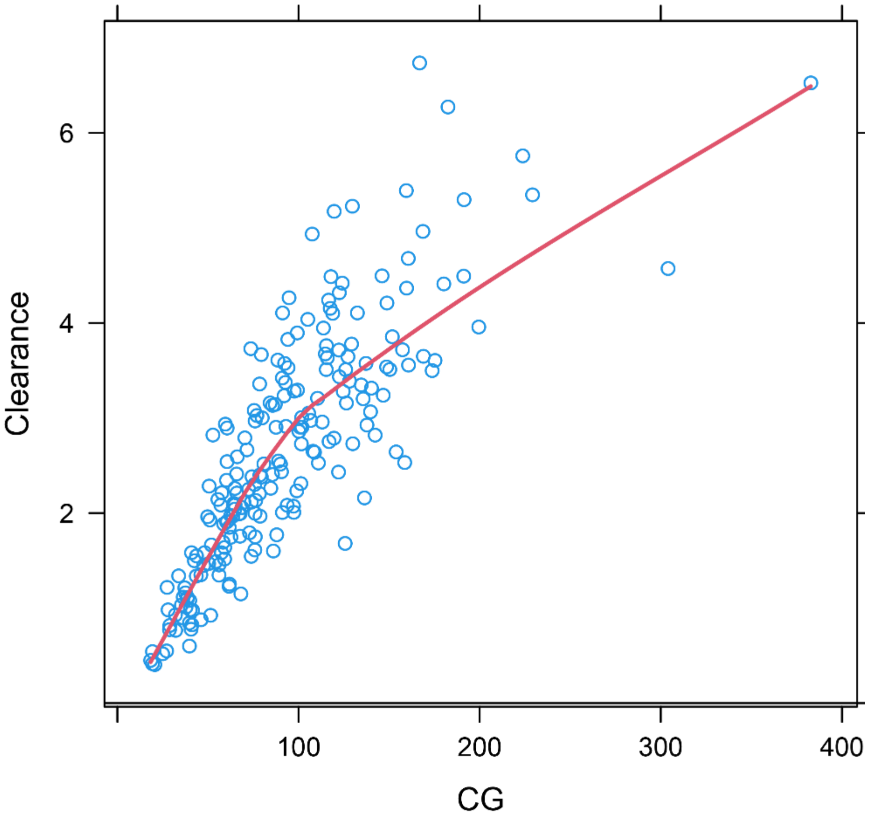

Figure S3. Scatter diagram format with creatinine clearance calculated by the Cockcroft–Gault equation (CG) and the drug clearance (Clearance). Blue circles: estimated vancomycin clearance. Red solid line: LOESS line.

Table S1. Recommended initial dosage regimens for targeted trough concentration of 10-20 mg/L.

CL_{cr} (mL/min).	Non-ICU patients		ICU patients	
	**Dosage**	**PTA (%)**	**Dosage**	**PTA (%)**
15-29	250 mg Q24 h	54.46	250 mg Q24 h	50.40
30-44	250 mg Q12 h	56.03	250 mg Q12 h	67.49
45-59	500 mg Q12 h	54.61	250 mg Q8 h	64.07
60-89	500 mg Q8 h	50.85	500 mg Q12 h	56.09
90-119	750 mg Q8 h	50.03	500 mg Q8 h	57.08
120-149	1500 mg Q12 h	49.42	500 mg Q8 h	55.70
150-179	1750 mg Q12 h	49.12	750 mg Q8 h	51.48
≥180	2000 mg Q12 h	48.53	750 mg Q8 h	53.62

CL_{cr}, creatinine clearance; PTA, probability of steady-state trough concentration between 10 and 20 mg/L attainment.
